# Neutrophil extracellular traps are present in the airways of ENaC-overexpressing mice with cystic fibrosis-like lung disease

**DOI:** 10.1186/s12865-021-00397-w

**Published:** 2021-01-21

**Authors:** Samantha L. Tucker, Demba Sarr, Balázs Rada

**Affiliations:** Department of Infectious Diseases, College of Veterinary Medicine, The University of Georgia, Athens, GA USA

**Keywords:** Cystic fibrosis, Neutrophil extracellular traps, NET, ENaC, neutrophil

## Abstract

**Background:**

Neutrophils are key components of the exacerbated inflammation and tissue damage in cystic fibrosis (CF) airways. Neutrophil extracellular traps (NETs) trap and kill extracellular pathogens. While NETs are abundant in the airways of CF patients and have been hypothesized to contribute to lung damage in CF, the in vivo role of NETs remains controversial, partially due to lack of appropriate animal models. The goal of this study was to detect NETs and to further characterize neutrophil-mediated inflammation in the airways of mice overexpressing the epithelial sodium channel (βENaC-Tg mice on C57BL/6 background) in their lung with CF-like airway disease, in the absence of any apparent bacterial infections.

**Methods:**

Histology scoring of lung tissues, flow cytometry, multiplex ELISA, immunohistochemistry and immunofluorescence were used to characterize NETs and the airway environment in uninfected, βENaC-Tg mice at 6 and 8 weeks of age, the most chronic time points so far studied in this model.

**Results:**

Excessive neutrophilic infiltration characterized the lungs of uninfected, βENaC-Tg mice at 6 and 8 weeks of age. The bronchoalveolar lavage fluid (BALF) of βENaC-Tg mice contains increased levels of CF-associated cytokines and chemokines: KC, MIP-1α/β, MCP-1, G-CSF, IL-5, and IL-6. The BALF of βENaC-Tg mice contain MPO-DNA complexes, indicative of the presence of NETs. Immunofluorescence and flow cytometry of BALF neutrophils and lung tissues demonstrated increased histone citrullination, a NET-specific marker, in βENaC-Tg mice.

**Conclusions:**

NETs are detected in the airways of βENaC-Tg mice, in the absence of bacterial infections. These data demonstrate the usefulness of the βENaC-Tg mouse to serve as a model for studying the role of NETs in chronic CF airway inflammation.

**Supplementary Information:**

The online version contains supplementary material available at 10.1186/s12865-021-00397-w.

## Background

Cystic fibrosis (CF) is an autosomal recessive disease characterized by pancreatic insufficiency and chronic airway inflammation and infection.

In CF, reduced mucus clearance and chronic infection result in a constant cascade of immune cell recruitment and inflammation in the lung [1–3]. Neutrophils are recruited to sites of infection where they kill pathogens by a number of mechanisms, including phagocytosis, oxidative burst and degranulation [4]. Neutrophils also kill pathogens by the formation of neutrophil extracellular traps (NETs). NETs are web-like structures of DNA, histones and granule proteins expelled from neutrophils that entrap and kill extracellular pathogens [4, 5]. Key enzymes in the formation of NETs include neutrophil elastase, that degrades intracellular proteins and triggers nuclear disintegration, and peptidyl arginine deiminase type 4 (PAD4) that citrullinates histones to facilitate the nuclear decondensation and the release of chromosomal DNA [6–9]. While NETs function to control extracellular pathogens, excessive NET formation and subsequent release of DNA, histones and granule proteins in the environment can trigger tissue damage and organ dysfunction [3, 10, 11].

Exuberant NET release has been proposed in CF airways [11–13]. We have demonstrated that both laboratory isolates and CF clinical isolates of *Pseudomonas aeruginosa*, a key bacterial agent of CF lung infections and one of the most important pathogens in progressive and severe CF lung disease, strongly trigger NET release [14–16]. Both DNA and NETs have been detected in the lungs of CF patients [11–13]. Free DNA in CF airways has been correlated with reduced lung function, as well as increased levels of neutrophil-recruiting chemokines, and risk of infection [12]. We have also recently provided evidence that adult CF patients develop an autoimmune response against NET components that correlates with worsening of lung disease [17]. While NETs are abundant in CF airways, they fail to clear respiratory pathogens and were hypothesized to contribute to lung damage in CF, the exact mechanistic role of NETs in CF airway disease pathogenesis remains unclear. This is partially due to lack of studies investigating NETs in CF animal models.

While several mammalian species are currently used in biomedical research to study CF lung disease, the advantages of the mouse as a disease model are unparalleled due to the availability of reagents and several transgenic strains, tools essential for detailed mechanistic studies of biological processes. Unfortunately, *Cftr*-deficient mice also express a CFTR-independent alternative chloride channel and still secrete chloride, thereby compensating for their dysfunctional CFTR [18]. Additionally, these mice fail to develop CF-like lung disease [18–20]. On the other hand, transgenic mice with airway epithelial cell-specific overexpression of the beta subunit of the *Scnn* gene that codes for the beta subunit of the epithelial sodium channel (βENaC) were generated [19, 21, 22]. ENaC is responsible for Na^+^ absorption in the airway epithelium and is upregulated in CF patients [21]. Studies of these mice (βENaC-Tg) demonstrated that increased airway sodium absorption causes airway surface liquid depletion, reduced mucus transport, and spontaneous CF-like lung disease with airway mucus obstruction, impaired mucociliary clearance, emphysema, and chronic inflammation including airway neutrophilia, similar to human CF lung disease [19, 21–23]. *P. aeruginosa* can infect the airways of βENaC-Tg mice, both in planktonic and biofilm forms, and form bacterial aggregates [23, 24], a well-described feature of *P. aeruginosa* in CF patients with chronic infection [25–28]. The goal of this work was to characterize NETs and further details of the neutrophil-mediated inflammation in the airways of βENaC-Tg mice, as the best murine CF lung disease model, in the absence of bacterial infections. We chose later time points (6 and 8 weeks) than any study using this model before to better represent established, chronic lung disease.

## Methods

### Mice

All animal procedures were approved by the Animal Care and Use Committee at the University of Georgia. The βENaC-Tg mouse line on a C57BL/6 genetic background was obtained from Alessandra Livraghi-Butrico (University of North Carolina, Chapel Hill, NC) with the permission of Marcus Mall (Charité - Universitätsmedizin Berlin, Germany) who generated this model. This mouse strain is also available at Jackson Laboratory: B6.Cg-Tg (Scgb1a1-Scnn1b)6608Bouc/J, (Stock No: 006438). Generation of the transgenic mouse was described previously [21]. Mice heterozygous positive for the overexpressing transgene were confirmed by PCR using forward primer (P1) 5′-CTTCCAAGAGTTCAACTACCG-3′ and reverse primer (P2) 5′-TCTACCAGCTCAGCCACAGTG-3′ to amplify the intron region of *Scnn1b*. Expected sizes were 254 bp for βENaC-Tg and ~ 350 bp for wild-type (Supplemental Figure 1). Adult βENaC-Tg mice on a C57BL/6 background were studied at 6 and 8 weeks of age, and wild-type (WT), same age C57BL/6 littermates served as controls. All mice in this study had free access to food and water. Mice were bred as hemizygotes. Animals were randomly allocated to experimental groups and randomly assessed. Mice were anesthetized with tribromoethanol TBE (IP; 180–250 mg/kg) administered one time per mouse intraperitoneally. A negative response to a toe pinch confirmed adequate anesthetic depth. Animals were euthanized with CO_2_ and subsequent cervical dislocation. Equal numbers of male and female mice were included into the study. The weight of animals at time of their euthanasia was: wild-type male (24.0 +/− 3.7 g), wild-type female (19.3+/− 1.4 g), βENaC-Tg male 23.5 +/− 2.7 g), βENaC-Tg female (19.0 +/− 0.9 g) (mean+/−S.D.). Mice were studied at 6 and 8 weeks of age. For identification purposes, standard ear-tagging and tail-clipping was done. Mice were maintained by breeding on campus in the UGA Veterinary Medical College Central Animal Facility rodent vivarium. Pups were weaned from the mothers at a standard 21 days unless they appeared unable to support themselves, in which case they were weaned at 28 days. The mice were kept in microisolator cages. After euthanasia, lungs were either subjected to bronchoalveolar lavage fluid collection or to fixation followed by pathological examination, immunohistochemistry or immunofluorescence staining. No experimental drugs were used in this study.

### Histopathology evaluation

Murine lungs were collected at 6 and 8 weeks of age from uninfected animals and were inflated with 1 mL of 10% Neutral Buffered Formalin. Tissue sections were prepared by the Histology Laboratory at the College of Veterinary Medicine at UGA. Sections were either left unstained for subsequent experiments or stained with hematoxylin and eosin (H&E). H&E slides from both C57BL/6 and βENaC-Tg mice were analyzed by a pathologist who was blinded to the experiments (*n* = 8–13 mice per group). Lesions and severity of lesions in the bronchioles, alveoli, interstitium, blood vessels, and pleura were assessed. Immune cell recruitment was also assessed. A scale from 0 to 4 was given to the severity of lesions with 0 indicating histologically normal, 1 minimal, 2 mild, 3 moderate, and 4 marked.

### Immunohistochemistry

For immunohistochemistry with 3,3′-Diaminobenzidine (DAB) amplification of signal, paraffin-embedded lung tissue unstained sections from uninfected WT and βENaC-Tg mice were deparaffinized and rehydrated with xylene and alcohol gradient. Tissue sections were antigen-retrieved with 0.1 M sodium citrate in Pascal Pressure Cooker (DakoCytomation, Aligent Technologies, Santa Clara, CA) for 20 min at 95 °C. Antigen retrieval was followed by endogenous peroxidase blocking with Dual Endogenous Enzyme Block (Dako, Cat# S2003). Sections were then incubated in a blocking buffer (10% Normal Goat Serum) followed by incubation with primary antibody against neutrophil elastase (1:2000 Abcam, Cat# 21595) overnight. After washing with 1X TBS/0.5% Tween, sections were incubated with polymer HRP (GBI Labs, Cat# D13–18) followed by 5 min DAB treatment. Hematoxylin (Vector Laboratories, Cat# H3401) and Acrytol Mounting Medium (EMS, Cat# 13158) were used for counterstaining and mounting, respectively. Pictures were taken with an Olympus BX41 phase contrast and dark field microscope, and analyzed with cellSens Entry software (Olympus, Center Valley, PA).

### Flow cytometry

To characterize myeloid cell subsets, bronchoalveolar lavage fluid (BAL) was collected from adult, uninfected βENaC-Tg and WT mice at 6 and 8 weeks of age (*n* = 6/group). The airways of euthanized mice were washed with 1 ml of sterile 1X PBS. Retained BAL was centrifuged at 400 x g for 10 min. The cells recovered from the wash were suspended in 1 ml sterile 1X PBS, counted and stained to detect the number of cells from each of the following myeloid cell subsets: neutrophils (CD11b^+^, CD115^−^, Ly6G^+^), eosinophils (CD11b^+^, CD115^−^, CD11c^−^, Ly6G^−^, Ly6c^lo^); monocytes (CD11b^+^, CD115^+^, Ly6G^+^); inflammatory monocytes (CD11b^+^, CD115^+^, Ly6G^High^), macrophages (CD11b^+^, F4/80^+^): dendritic cells (CD11b^+^,CD11c^+^,F4/80^−^); alveolar macrophages (CD11b^+^, F4/80^−^, CD115^+^, CD11c^−^) and inflammatory macrophages (CD11b^+^, F4/80^+^, CD115^+^, CD11c^+^). All antibodies were purchased from Biolegend (San Diego, CA). Cells were suspended in 100 μl PBS and incubated with Zombie Aqua fixable viability dye (1:1000, Biolegend, San Diego, CA) at room temperature in the dark for 15 min to distinguish between live and dead cells collected from the BAL. After centrifugation, cells were resuspended in 100 μl PBS/1% BSA. All subsequent cell processing was performed on ice, protected from light. Cells were blocked with TruStain FcX™ (Biolegend, San Diego, CA) for 10 min. All antibodies were added to the cells at a 1:100 dilution at the same time. Cells were incubated for 1 h, then washed with PBS/1% BSA and re-suspended in 500 μl Stabilizing Fixative (BD Biosciences, San Jose, CA), and stored at 4 °C until analysis. Samples were read at the University of Georgia College of Veterinary Medicine Cytometry Core Facility on a BD LSRII flow cytometer (BD Biosciences, San Jose, CA) within 24 h of staining. Data were analyzed with the BD FACsDiva™ software (BD Biosciences, San Jose, CA). The gating strategy is shown in Supplemental Figure 2.

To measure the amount of histone citrullination occurring in the airways of adult βENaC-Tg and WT mice at 6 and 8 weeks of age, BAL was harvested as described above. Within each group, BALs were pooled to make a total of 1 × 10^6^ cells/sample (*n* = 5–6/group). Citrullinated histone H3-positive neutrophils were defined as CD11b^+^, CD115^−^, Ly6G^+^, histone H3 (citrulline R2+R8+R17)^+^. The gating strategy is shown in Supplemental Figure 4. Cells were suspended in 100 μl PBS and incubated with Zombie Aqua fixable viability dye (1:10,000, Biolegend, San Diego, CA) at RT in the dark for 15 min to distinguish between live and dead cells collected from the BAL. Cells were washed with PBS/1% BSA, and then fixed and permeabilized with the Fix & Perm/ Cell Fixation and Permeabilization Kit (Abcam, Cambridge, MA) following manufacturer’s instructions. All subsequent cell processing was performed on ice, protected from light. Cells were blocked with TruStain FcX™ (Biolegend, San Diego, CA) for 10 min. The primary anti-histone H3 (citrulline R2+R8+R17) (Abcam, Cambridge, MA) antibody was incubated with the cells for 30 min. Cells were washed with PBS/ 1% BSA followed by incubation with donkey-anti-rabbit-PE (Biolegend, San Diego, CA) for 30 min. Cells were washed with PBS/ 1% BSA. The conjugated antibodies for CD11b, CD115, Ly6G (Biolegend, San Diego, CA) were added together and incubated for 30 min. Cells were washed with PBS/1% BSA and resuspended in 500 μl Stabilizing Fixative (BD Bioscience, San Jose, CA), and stored at 4 °C until analysis. Flow cytometry was performed at the University of Georgia CTEGD Shared Resources Laboratory on a Cyan ADP cytometer (Beckman Coulter, Hialeah, Florida, NIH grant # 1S10RR027814) within 24 h of staining. Data were analyzed using FlowJo™ Software for Windows, version 10 (Becton Dickinson and Company, Ashland OR).

### Immunofluorescence assay

Cells were harvested from the BAL of adult βENaC-Tg and WT mice at 6 and 8 weeks of age as described above. 1.5X10 [5] cells, pooled from multiple mice/cohort were adhered to precleaned microscope slides using Double Cytofunnel Sample Chambers (Thermo Scientific, Waltham, MA) and a cytospin preparations were performed with a Shandon Cytopsin 2 centrifuge (Thermo Scientific, Waltham, MA). Cells were fixed with 4% paraformaldehyde for 15 min and washed with PBS. Cells were permeabilized with 0.1% Triton X100 while blocking in PBS with 5% BSA and 10% goat or horse serum for 1 h. Primary antibody staining to detect myeloperoxidase (RD Systems, Minneapolis, MN) and/or histone H3 (citrulline R2 + R8 + R17) (Abcam, Cambridge, MA) was performed at a 1:250 dilution in PBS with 1% BSA, 1% goat or horse serum overnight at 4C. Slides were washed in PBS. Secondary antibody staining was performed at a 1:500 dilution in PBS with 1% BSA, 1% goat or horse serum for 1 h with horse- anti-rabbit IgG Dylight™ 488 for citrullinated histone, or horse-anti-goat Dylight™ 594 for myeloperoxidase (Vector Laboratories, Burlingame, CA). Slides were washed with PBS. Vectashield™ anti-fade mounting medium with DAPI (Vector Laboratories, Burlingame, CA) was applied to the cells prior coverslip addition. For immunofluorescence assay of histology sections, paraffin-embedded lung tissues were de-paraffinized as described above. Antigen unmasking and antibody staining was performed following the protocol described by Abed and Brinkmann, 2019 [29]. Both the myeloperoxidase (RD Systems, Minneapolis, MN) and histone H3 (citrulline R2 + R8 + R17) (Abcam, Cambridge, MA) primary antibodies were used at 1:500 dilution. Secondary antibody staining was performed at a 1:1000 dilution in PBS with 1% BSA, 1% goat or horse serum for 1 h with horse-anti-rabbit IgG Dylight™ 488 for citrullinated histone, or horse-anti-goat Dylight™ 594 for myeloperoxidase (Vector Laboratories, Burlingame, CA). Vector® Laboratories TrueView® autofluorescence quenching kit (Vector Laboratories, Burlingame, CA) was used prior to coverslip addition. All digital images were acquired at the University of Georgia College of Veterinary Medicine Cytometry Core on a Nikon A1R confocal microscope (Nikon Eclipse Ti-E inverted microscope) and examined with NIS Element software (Nikon, Version 6.4).

### MPO-DNA ELISA

Complexes of MPO-DNA were detected in the BAL of adult βENaC-Tg and WT mice at 6 and 8 weeks of age using a published protocol adapted to murine samples [15, 30]. Supernatants from the BAL of mice (*n* = 6/group) were diluted 1:50 in PBS. Diluted samples were added to a 96-well plate, pretreated overnight at 4 °C with capture anti-MPO (1:200 RD Systems, Minneapolis, MN), and blocked with 5% BSA in PBS at RT for 2 h. Samples were incubated overnight at 4 °C. Following 3X washes with PBS/Tween 20, the secondary anti-DNA-POD (1:500, Roche, Basal, Switzerland) was added for 1 h at room temperature. Samples were washed 4X with PBS/Tween 20. TMB substrate (Thermo Scientific, Waltham, MA) was added. The reaction was stopped by the addition of a 1 M HCl solution. Absorbance was measured at 450 nm with an Eon microplate spectrophotometer (BioTek, Winooski, VT). Background absorbance values of the medium and untreated neutrophils were subtracted. All samples being compared were run on the same plate in two trials. Differences between optical densities were compared.

### Bioplex cytokine array

Soluble protein analytes of mouse BAL supernatants from 6 and 8 week-old βENaC-Tg mice and WT mice (*n* = 9/group) were preserved with HALT™ protease inhibitor cocktail (Thermo Scientific, Waltham, MA) before storage at − 80 °C. Bead-based multiplexing was used to measure BAL levels of 23 different cytokines and chemokines using internal standards (Bioplex Pro-Mouse Cytokine Grp1 Panel, Cat# M60009RDPD) following manufacturer’s instructions (Bio-Rad Inc., Hercules, CA). The assay was performed at the University of Georgia CTEGD Shared Resources Laboratory on a Bio-Plex analyzer (Bio-Rad Inc., Hercules, CA).

### Statistical analysis

Results between two groups were analyzed by Student’s t-test and data among more than two cohorts were compared by One-Way ANOVA, or the nonparametric Kruskal-Wallis test. Analysis of data sets comparing more than two variables for more than two cohorts were analyzed using a Two-Way ANOVA. Data are expressed as mean plus-minus standard error of the mean (SEM). Statistically significant differences were considered as *, *p* < 0.05; **, *p* < 0.01; ***, *p* < 0.001. Exact *p* values for each test are listed in the figure legends. Statistical analysis was carried out with GraphPad Prism version 6.07 for Windows software.

## Results

### Lung pathology in 8 week-old β-ENaC-Tg mice

**Fig. 1:**
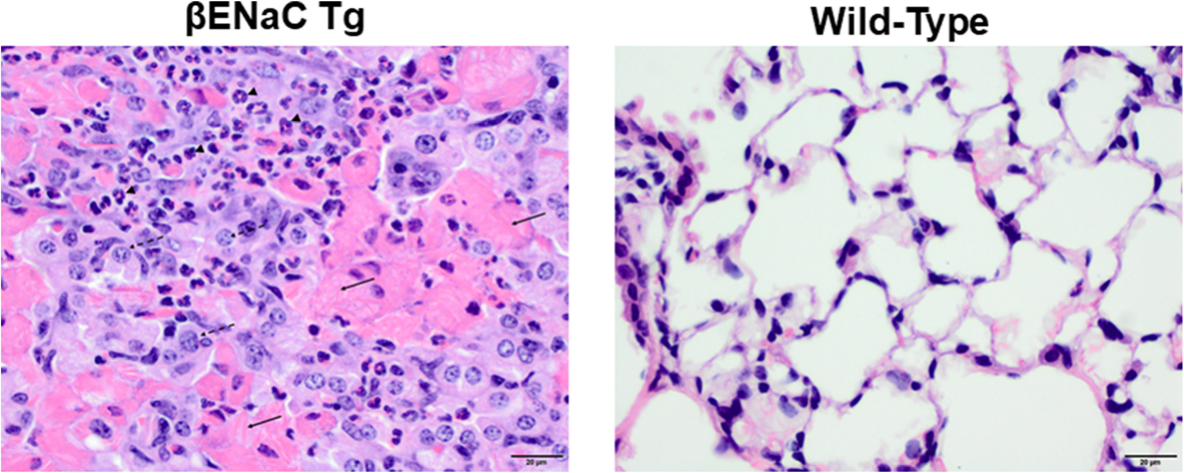
Clinical lung pathology in 6- and 8-week old βENaC-Tg mice. Paraffin embedded histological lung sections from βENaC-Tg (left) and wild-type (right) mice at 6 and 8 weeks were stained with hematoxylin and eosin. Some of the hypereosinophilic areas exhibit crystal-like or needle-like arrangements (solid arrows) and are surrounded by a mixture of neutrophils (arrowheads) and macrophages (large cells with round or slightly indented nucleus and abundant eosinophilic cytoplasm; dashed arrows) in large numbers. Representative images shown of six similar results. Hematoxylin and eosin staining, 400x original magnification, scale bar = 20 μm

To characterize lung pathology in βENaC-Tg mice at the ages of 6 and 8 weeks, hematoxylin and eosin staining was performed on fixed lung tissue sections (*n* = 8–13 mice per group) (Fig. 1). Heavy inflammatory infiltrates characterize βENaC-Tg lung tissues (Fig. 1.) It was also noted that many of the βENaC-Tg mice had acidophilic macrophage pneumonia and eosinophilic crystal accumulation (Fig. 1). This observation is consistent with earlier studies characterizing the βENaC-Tg mouse lung environment at earlier time points [22, 31].

### Increased neutrophilic recruitment in the lungs of 8 week-old β-ENaC-Tg mice

**Fig. 2:**
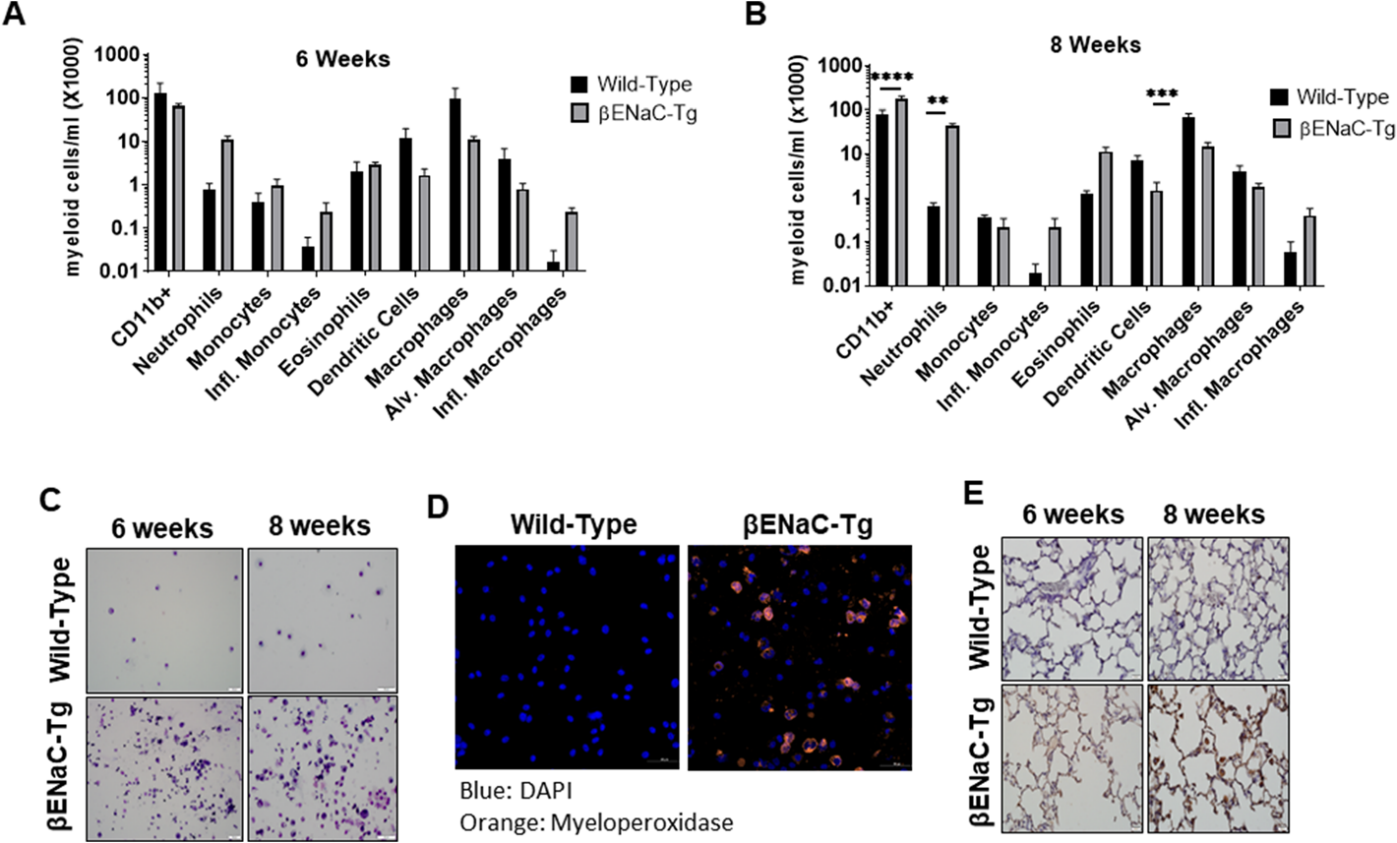
Neutrophil-dominated inflammation in the airways of βENaC-Tg mice at 6 and 8 weeks. Flow cytometry was used to measure myeloid cell populations collected from the BAL of mice at (**a**) 6 and (**b**) 8 weeks. There were significantly more myeloid cells, and neutrophils per 1000 cells in the BAL of βENaC-Tg mice at 8 weeks, as compared to wild-type mice. Statistical significance was determined with a Two-way ANOVA (*n* = 6, **, *p* < 0.05, ***, *p* < 0.001, ****, *p* < 0.0001). **c** Cytospin preparations of BAL from representative WT or βENaC-Tg mice were fixed and stained to visualize the cellular makeup of the BAL. **d** Immunofluorescence staining of 1.5 × 10^5^ cells from 1 to 2 mice per group shows neutrophils in the BAL of 8 week-old βENaC-Tg mice through detection of mouse myeloperoxidase (MPO, orange) and DNA (blue, DAPI). **e** Immunohistochemistry to detect neutrophil elastase was performed on paraffin-embedded lung tissue sections from mice at 6 and 8 weeks of age. Shown are representative images from each group of at least six similar results

To characterize leukocyte infiltration in the lungs of βENaC-Tg mice at the age of 8 weeks, flow cytometry was performed to assess myeloid cell populations in the BAL using surface markers to distinguish among neutrophils, monocytes, inflammatory monocytes, eosinophils, dendritic cells, macrophages, alveolar macrophages, and inflammatory macrophages (Supplemental Figure 2). BAL was collected from uninfected mice at 6 and 8 weeks of age (*n* = 6 per group), and the number of cells/ml was determined for each cell type listed above. While there was no significant difference in cell populations present in βENaC-Tg and WT mice at 6 weeks of age, there was a trend towards higher numbers of neutrophils and inflammatory macrophages in transgenic animals (Fig. 2a). This trend became significant in 8 week-old mice as there were 2 times more total myeloid cells (*p* < 0.0001) and 66 times more neutrophils in the βENaC-Tg mice compared to WT (*p* = 0.0049) (Fig. 2b). Most likely the enhanced neutrophilic recruitment accounted for most of the overall increase in total myeloid cell counts. Additionally, there was a significant 4.5-fold decrease in total macrophages present in the BAL of the βENaC-Tg mice compared to WT animals (*p* = 0.0002) (Fig. 2b). To demonstrate the increased presence of myeloid cells, inflammation and tissue damage in the lungs of the βENaC-Tg animals, cytospin preparations of equal volumes of BAL fluids followed by staining with Hema 3 stain™ were generated from mice in each group (Fig. 2c). These results show increased number of cells and inflammatory debris in the βENaC-Tg BAL fluid compared to an equal volume of BAL fluid from WT mice at 6 and 8 weeks, indicating heavy ongoing inflammation. Additionally, while –as expected- macrophages dominate in the lungs of WT mice, neutrophils are the dominant cell type in the airways of βENaC-Tg animals (Fig. 2c). To further confirm neutrophil dominance in the βENaC-Tg mouse lung, immunofluorescence staining of myeloperoxidase (MPO) of equal cell counts (1.5 × 10^5^) from BAL of both mouse strains shows the increased presence of neutrophils in the lungs of the βENaC-Tg mice (Fig. 2d). Lastly, immunohistochemistry for neutrophil elastase (NE) shows increased neutrophil recruitment in the airways of βENaC-Tg mice at both 6 and 8 weeks of age (Fig. 2e). These results reporting neutrophil-dominant leukocyte infiltration in the lungs of βENaC-Tg animals at later time points (6 and 8 weeks) are consistent with previous characterizations of the βENaC-Tg mouse model at earlier time points and with human CF lung disease [3, 19, 21].

### Neutrophil-recruiting cytokines are elevated in βENaC-Tg animals at 6 and 8 weeks of age

**Fig. 3:**
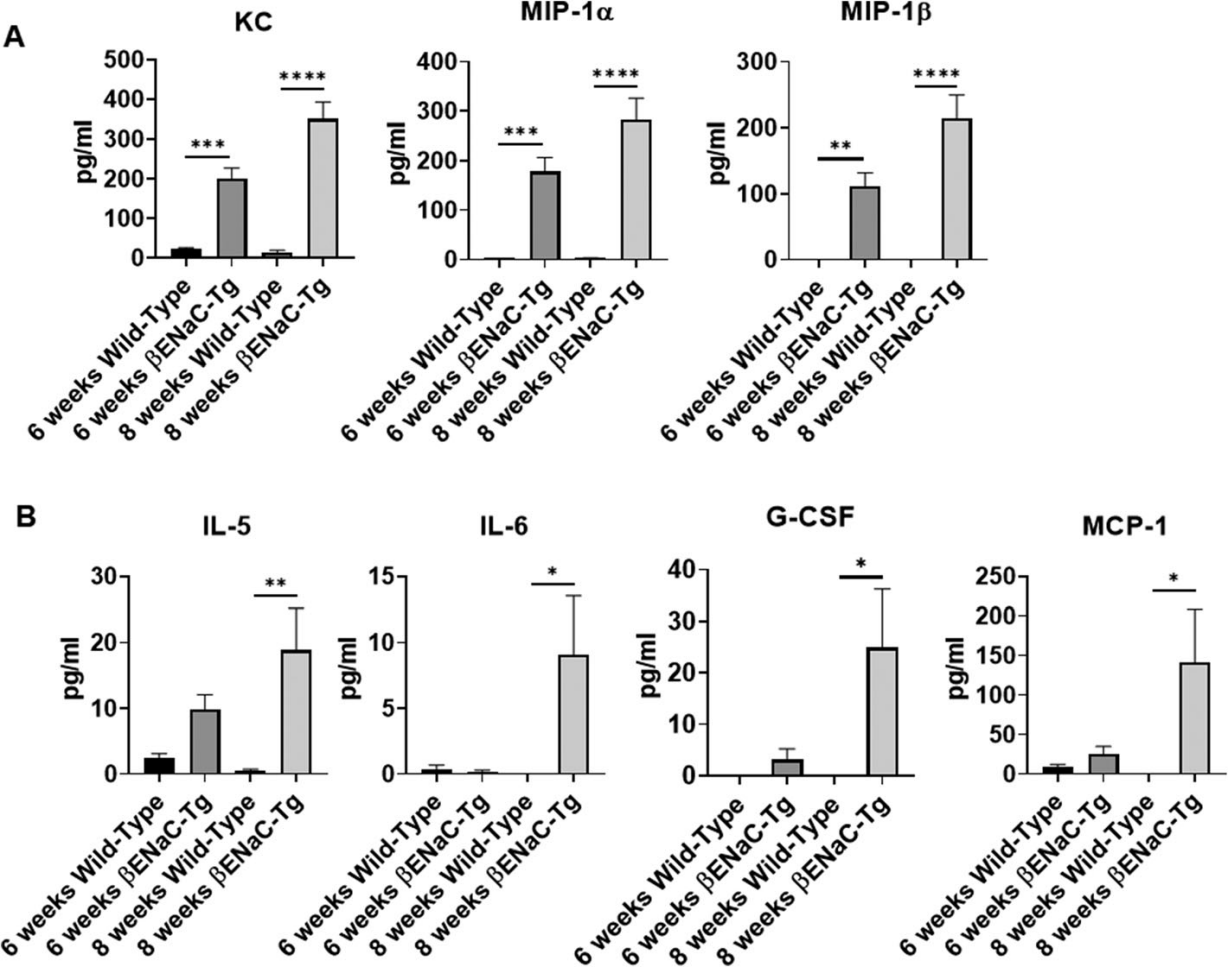
Neutrophil-recruiting cytokines are elevated in βENaC-Tg animals at 6 and 8 weeks of age. Bead-based multiplex array was used to analyze 23 cytokines and chemokines from BAL supernatants of βENaC-Tg mice and WT controls at 6 and 8 weeks of age. **a** Analysis shows significantly more KC, MIP-1α, and MIP-1β in both 6 and 8 week-old βENaC-tg mice as compared to wild-type. **b** There were significantly higher amounts of IL-5, IL-6, G-CSF, and MCP-1 detected in the BAL of 8 week-old βENaC-Tg mice compared to WT controls. One-way ANOVA was used to determine statistical significance (*n* = 9, *,= *p* < 0.05, **, *p* < 0.01, ***, *p* < 0.001, ****, *p* < 0.0001)

PMNs are driven to the airway space by chemoattractants. In CF, several PMN-guiding cytokines and chemokines were shown to be elevated. To explore the cytokine levels in the BAL of βENaC-Tg mice, a bead-based, multiplex cytokine/chemokine array measuring 23 target molecules was applied. The results of all analytes measured are shown in Supplemental Figure 3. Data showed significant increases ranging from 9 to 200-fold in neutrophil-attracting chemokines, KC, MIP-1α and MIP-1β in βENaC-Tg at both 6 and 8 weeks of age (*p* < 0.006, *p* = 0.0006, *p* < 0.0001 (Fig. 3a). Additionally, BAL levels of the proinflammatory cytokines IL-5 and IL-6, the monocyte chemoattractant MCP-1, and the neutrophil proliferation cytokine G-CSF were significantly elevated by 44-, 9-, 140-, and 25-fold, respectively, in β-ENaC-Tg mice compared to control animals at 8 weeks of age (*p* = 0.03, *p* < 0.006) (Fig. 3b). These data suggest that βENaC-Tg mice develop chronic airway inflammation characterized by elevated levels of cytokines known to be also increased in human CF patients.

### Neutrophil extracellular traps are present in the lungs of βENaC-Tg mice

**Fig. 4:**
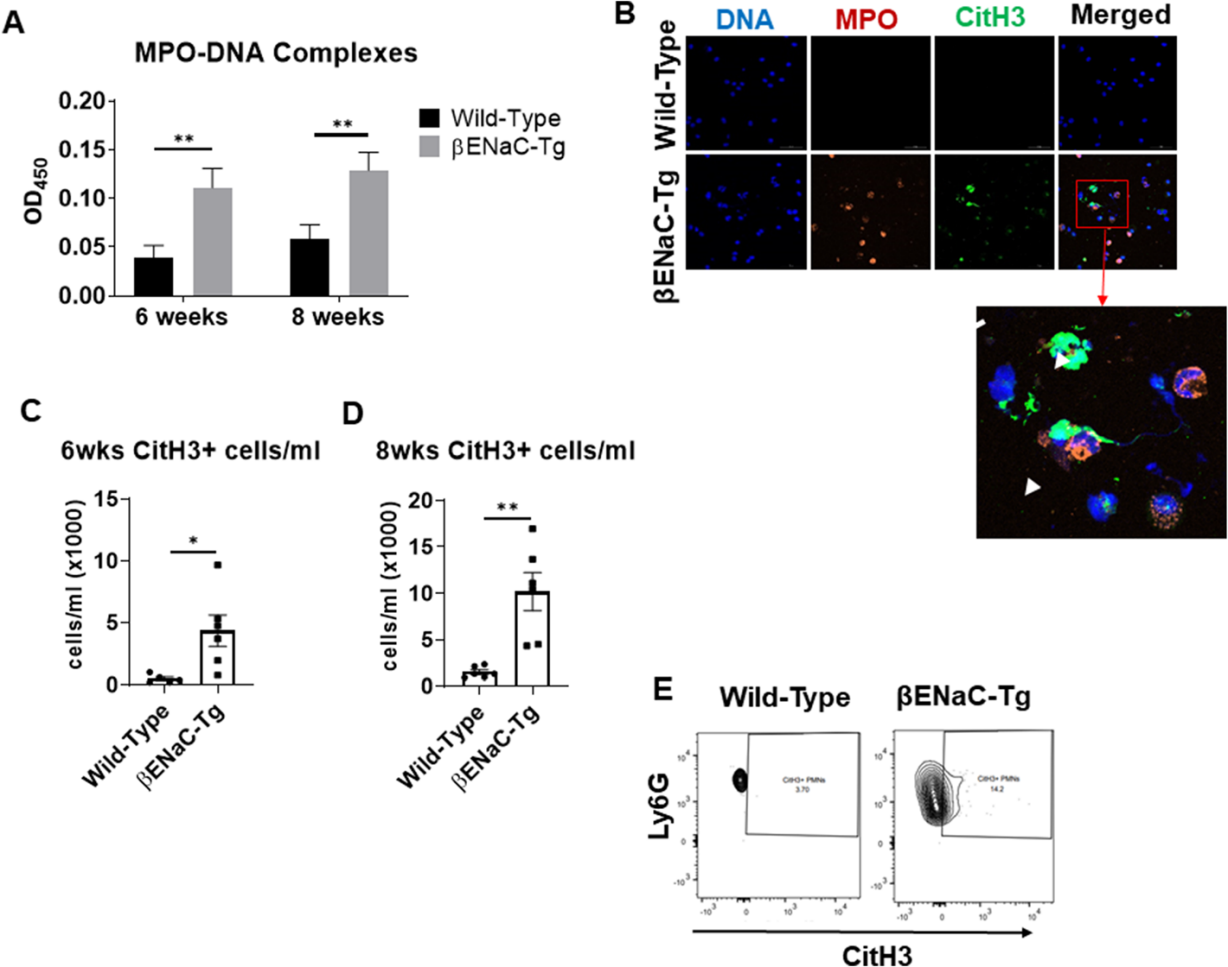
NETs are present in the bronchoalveolar lavage fluid of βENaC-Tg mice. **a** ELISA was used to detect MPO-DNA complexes, indicative of NETs, in BAL supernatants of mice at 6 and 8 weeks. There were significantly more MPO-DNA complexes detected in the BAL supernatant of βENaC-Tg mice than WT at both 6 and 8 weeks of age. One-way ANOVA was used to determine statistical significance (*n* = 6, **, *p* < 0.01). **b** Immunofluorescence imaging of 1.5 × 10^5^ cells from 1 to 2 mice per group was performed to detect NETs in the BAL of 8 week-old mice. Images are representative of 1 slide/ group. The larger image represents a closer view of the cells within the red square. The white arrows indicate NETs and the white arrow heads mark resting neutrophils characterized by distinct MPO and DNA staining patterns and lack of citrullinated histone staining. Blue=DAPI, orange=MPO, green=CitH3. Flow cytometry was used to quantify the number of cells undergoing histone citrullination in the BAL of (**c**) 6 and (**d**) 8-week-old mice. Pooled samples of 1 × 10^6^ cells from the BAL of 2–3 mice per group were assayed for citrullinated histone (CitH3) positive neutrophils. A Student’s t-test was used to determine statistical significance (*n* = 5–6/group, *, *p* < 0.05, **, *p* < 0.01). Citrullinated histone-positive neutrophils were considered as CD11b^+^/CD115^−^/Ly6G^+^/CitH3^+^. **e** Representative scatter plots showing CitH3^+^ neutrophils (Ly6G^+^)

Although the βENaC-Tg mouse model has been proposed as a model for CF airway disease, no study has investigated whether NETs are formed in the airways of these mice. To explore this, cell-free supernatants of BAL collected from βENaC-Tg mice and WT littermate controls were examined for the presence of MPO-DNA complexes, indicative of NETs, using an in-house generated ELISA assay as described [15, 30]. The absorbance values in BAL supernatants of βENaC-Tg mice were significantly higher than that of the WT littermate controls at both 6 and 8 weeks (Fig. 4a) indicating that NETs are present in the airways of βENaC-Tg animals.

### Increased histone citrullination in βENaC-Tg mice

To detect NETs by another method, immunofluorescence imaging of equal numbers of BAL cells isolated from both mouse strains was used. Co-localization of MPO, citrullinated histone 3 (CitH3), a hallmark of PAD4-dependent NET release [8], and DNA (DAPI) was observed that is indicative of NET release in the βENaC-Tg mouse (Fig. 4b). Only minimal MPO and CitH3 staining was detected in BAL cells from WT mice using the same microscope settings (Fig. 4b).

**Fig. 5:**
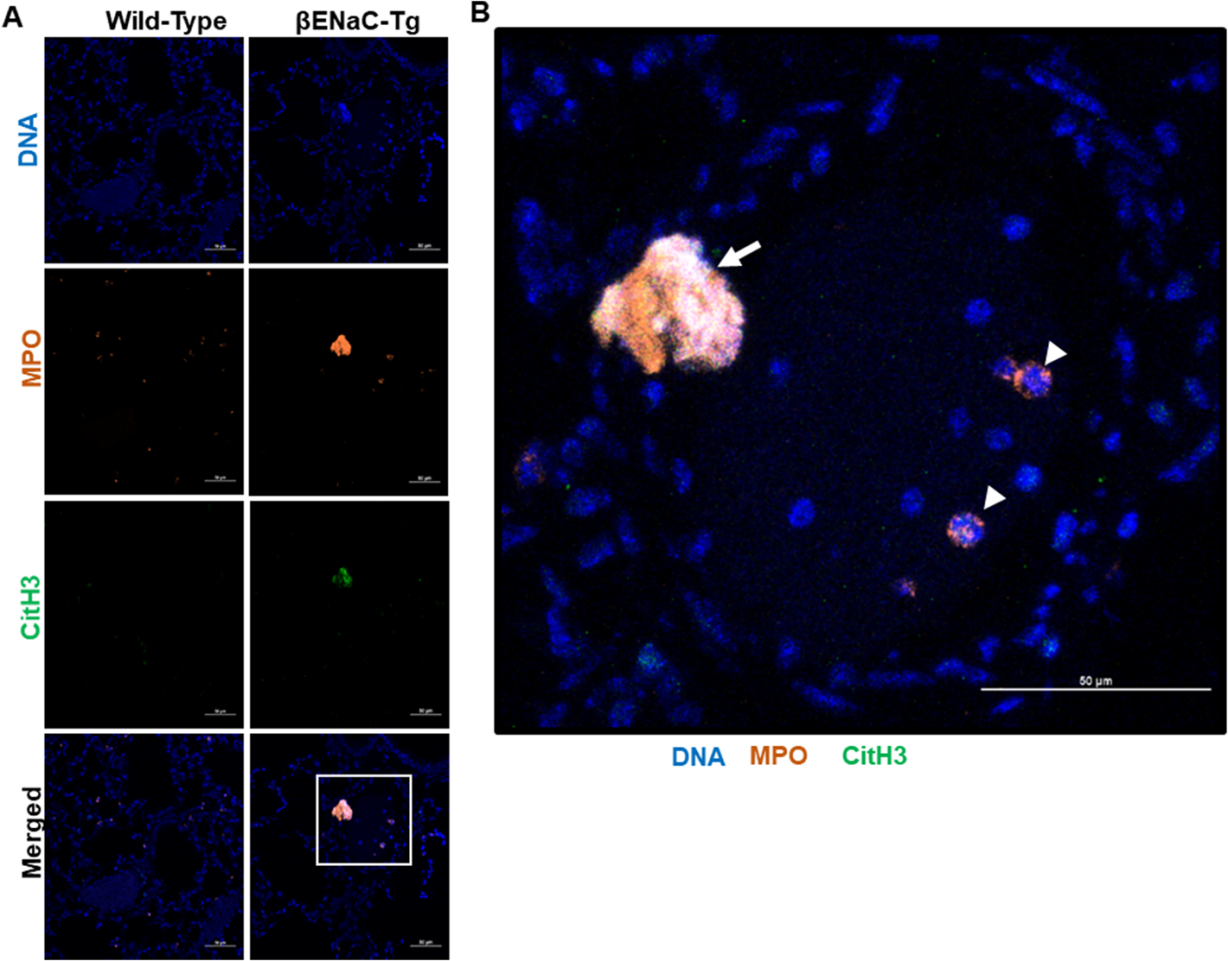
Neutrophil extracellular traps are present in the lungs of βENaC-Tg mice. **a** Immunofluorescence imaging of histology lung sections obtained from 8 week-old, wild-type and βENaC-Tg 8 mice (40X magnification). Co-localization of DNA (DAPI), MPO, and citrullinated histone 3 (CitH3) identify NETs. Blue=DAPI, orange=MPO, green=CitH3. Scale bars = 50 μm. **b** A more detailed view of the inset marked as a while square on the βENaC-Tg lung image shown in (**a**). The white arrow indicates NETs while the white arrow heads point to resting neutrophils characterized by distinct MPO and DNA staining patterns and lack of citrullinated histone staining. Images are representative of one section/ group. Scale bar = 50 μm

As a third approach to study NETs, flow cytometry was used to quantify the presence of neutrophils undergoing PAD4-mediated NET release. The gating strategy for this assay is shown in Supplemental Figure 4. The number of CitH3 positive neutrophils (CD11b^+^, CD115^−^, Ly6G^+^, CitH3^+^) was quantified using 1 × 10^6^ BAL cells, pooled from multiple mice from either βENaC-Tg or WT background at 6 and 8 weeks of age (Fig. 4c-e). There were significantly more CitH3 positive cells/ml in the BAL of βENaC-Tg mice at both ages compared to the WT controls. Lastly, immunofluorescence imaging of lung sections was performed on 8 week-old βENaC-Tg and WT mice. Granular, intracellular MPO staining, indicative of resting neutrophils, was detected in the tissues of both mouse strains (Fig. 5a). Large NETs marked by co-localization of MPO, citrullinated histone and DNA were detected in the lung of the βENaC-Tg mice but where absent in the lungs of their wild-type littermate controls (Fig. 5a and b). Overall, this data demonstrates the presence of NETs in the lungs of the βENaC-Tg mice.

## Discussion

The βENaC-Tg mouse model was generated in 2004, and was initially characterized as a model for studying CF by demonstrating the effects of increased Na^+^ secretion on airway liquid volume and mucus accumulation [21]. That study showed that overexpression of the β-subunit of ENaC led to decreased airway surface liquid, leading to mucus accumulation, and sterile, neutrophil-driven inflammation in the lungs of these mice by 4 to 6 weeks of age [21], a phenotype similar to human CF lung disease [3, 11]. Further characterization of the βENaC-Tg mouse confirmed these CF-like lung phenotypes including reduced mucus clearance, Goblet cell hyperplasia, neutrophilia, mucus plugging of the trachea, and Th2-mediated immune responses [22]. This study also demonstrated epithelial cell necrosis and signs of emphysema (lung volume, mean linear intercepts and destructive index), both common in human CF disease [22, 32, 33]. Another study demonstrated free DNA accumulation in the lungs of βENaC-Tg^12^. This accumulation correlated with both CXCL2 expression and reduced lung function, suggesting a link between free DNA and leukocyte infiltration in this model, similar to human CF^12^. A more recent study reported that βENaC-Tg mice can be infected with planktonic and biofilm forms of *P. aeruginosa*^24^ much better than wild-type animals, and the bacterium forms aggregates similar to those seen in human CF patients [25, 26]. Neutrophil elastase is also found in the airways of βENaC-Tg mice, similar to human CF patients including children [34], and was found to be a major contributor to lung disease pathogenesis [35]. These published reports overall confirm that βENaC-Tg mice serve as an appropriate model for CF lung disease.

No information is, however, available whether NETs form in the airways of βENaC-Tg animals. NETs and citrullinated histones are present in human CF airways [20, 36]. In the present study, we aimed at characterizing neutrophil-mediated inflammation and NETs in the airways of this mouse model at time points later than studied before to reveal whether it could serve as a mouse model for studying the role of NETs in established, chronic CF lung disease. Histological evaluation showed increased acidophilic macrophage pneumonia and eosinophilic crystal accumulation in the lungs 6 and 8 week-old βENaC-Tg mice. Previous characterizations have also shown this phenotype and confirmed the increase of chitinase-like proteins that lead to eosinophilic crystals in the lungs of these mice, as also in sputa and sera of CF patients [22, 31]. Others have also demonstrated histological pathologies in the βENaC-Tg mice that mirror human CF lung disease, including mucus plugs, airspace enlargement, inflammation, and mucin production [37]. Leukocyte phenotyping by flow cytometry examining nine different myeloid cell populations demonstrated significant and dominant neutrophil enrichment in the BAL of 8 week-old βENaC-Tg mice compared to littermate controls. While others have demonstrated increased neutrophils and eosinophils at early time points [19, 21], this is the first report to our knowledge detecting dendritic cells, monocytes, inflammatory monocytes, and multiple macrophage subtypes in the BAL of these mice. This observation was further confirmed through Hema 3 staining and MPO immunofluorescence staining of BAL cells, as well as immunohistochemistry detecting neutrophil elastase in paraffin-embedded tissue sections. These data reflect previous reports showing the value of the βENaC-Tg mouse model for studying neutrophil (NET)-driven airway inflammation in CF.

Chronic human airway disease is characterized by a hyper-inflammatory environment with enhanced levels of several cytokines and chemokines [38, 39]. A bead-based multiplex cytokine and chemokine array measuring 23 analytes showed significantly increased amounts of the neutrophil-recruitment chemokine KC (IL-8), as well as MIP-1α, and MIP-1β at 6 and 8 weeks in the BAL of βENaC-Tg mice. By 8 weeks of age, there was also significantly more IL-5, IL-6, G-CSF, and MCP-1 detected in the BAL of the βENaC-Tg mice compared to littermate controls. While other reports have previously shown increased levels of the major neutrophil chemoattractant KC in the BAL of βENaC-Tg mice at earlier time points [19, 21], here we report increased airway concentrations of MIP-1α, MIP-1β, MCP-1, IL-5, IL-6, and G-CSF in this CF mouse model. It has been reported that children with CF have higher levels of MCP-1, MIP-1α and MIP-1β in their BAL compared to healthy controls, regardless of bacterial infection status^40^. These chemokines play a role in recruiting activated macrophages to the site of infection, and may also contribute to the inflammation in CF airways [40]. ELISA measurements of sputum from adult CF patients during acute pulmonary exacerbation showed increased IL-6, G-CSF, MCP-1, and MIP-1β, all of which were still detected 10 days post-treatment [41]. Bead-based multiplex analysis of nasal lavage fluids from adult CF patients also showed detectable levels of IL-8, G-CSF, MCP-1, and MIP-1β both during pulmonary APE and following treatment [42]. Comparison of cytokine levels in the BAL of CF children with or without *P. aeruginosa* infection to healthy controls showed increased IL-6 levels regardless of infection status, and increased IL-5 concentration, which was further enhanced during infection [43]. This study also demonstrated increased IL-8 levels in bronchial epithelial tissues of CF patients, and showed that IL-5 and IL-8 concentrations correlate with lung damage imaged by computed tomography [43]. Lastly, this study demonstrated an increase in IL-5 secretion by CF T cells in response to *P. aeruginosa* stimulation [43]. Both IL-8 and IL-5 levels in nasal secretions have been positively correlated to neutrophil numbers in CF teenagers [44]. Additional reports from human studies have detected these cytokines and chemokines in other CF patient fluids, including tear fluid, plasma, and nasal lavage fluid [45–47]. The similarities in cytokine and chemokine profiles between human CF studies and our data in the βENaC-Tg mouse model demonstrate the usefulness of this model for studying lung inflammation in CF.

It has become evident that neutrophil-mediated inflammation is a major component of CF lung disease [4]. The increased interest in the role of neutrophils in CF demands a strong in vivo model to recapitulate human airway disease. The βENaC-Tg mouse has previously been presented as a model for CF lung disease to study mucus accumulation and neutrophilia [12, 19, 21]. These results are in contrast to data from the *Cftr*^−/−^ mouse, which does not present with airway obstruction or neutrophilia [19]. We further demonstrate the usefulness of this model for CF airway disease by showing NETs in the lungs of these mice, which is in line with human data [11, 12]. Neutrophil granule proteins like MPO and NE, as well as NET-derived extracellular DNA and NET components such as histones are found in the lungs of CF patients, and correlate with enhanced morbidity [12, 13]. Previous work in our laboratory has also shown that CF clinical isolates of *P. aeruginosa* stimulate NET formation and release of CF inflammatory markers [14, 15]. We have also recently shown a correlation between autoantibodies against the NET marker PAD4 and CF lung disease severity [17]. Others have shown reduced ability of CF NETs to effectively kill clinical isolates of *P. aeruginosa* [48]. More recently, studies of neutrophil function and regulation have shown delayed apoptosis, with a preference toward NET release in CF neutrophils [49], as well as evidence suggesting a more suppressory phenotype of CF neutrophils [50]. Here we show increased levels of MPO-DNA complexes, indicative of NETs, as well as evidence of PAD4-mediated NET release through both immunofluorescence assays and flow cytometry. This observation further supports the similarities in the airway environment between human CF patients and βENaC-Tg mice. Detection of NETs in βENaC-Tg mouse airways provides a platform for addressing the role of NETs in the inflammation and tissue damage in CF lung disease.

## Conclusions

Taken together, our data provide a comprehensive view of βENaC-Tg mouse airway inflammation at later, more chronic, time points further confirming the potential of this model for investigating CF lung disease. We have shown an innate immune cell environment, as well as a cytokine and chemokine profile that mirrors human CF disease. Additionally, we are the first to show the presence of NETs in the lungs of the βENaC-Tg mouse model. Neutrophils and NETs have been implicated in CF airway disease progression, and could represent a therapeutic target in CF. This work demonstrates the utility of the βENaC-Tg mouse for future studies aimed at addressing the role of neutrophils and NETs in CF lung inflammation and infection.

## Supplementary Information

**Additional file 1: Figure S1.** Genotyping of β-ENaC-Tg mice by PCR. Mice were identified as either WT C57BL/6 or heterozygous for over-expression of β-ENaC using PCR of the intron region of *Scnn1b*. (A) A schematic representation of the expected PCR product sizes for both WT and transgenic β-ENaC-Tg. (B) A representative gel electrophoresis comparing the final PCR product of WT and heterozygous β-ENaC-Tg mice. **Figure S2.** Flow cytometry gating strategy: myeloid cells. A representative schematic depicting the method for selecting myeloid cell populations present within the BAL of uninfected WT and β-ENaC-Tg mice. Single cells, negative for zombie aqua fixable viability dye were considered live. The myeloid cell marker CD11b was used to separate myeloid cells from other cell types. The CD11b+ cells were considered the parent cell population for all cell-types measured. The markers used for each cell type is as follows: Neutrophils (CD11b^+^, CD115^−^, Ly6G+), Eosinophils (CD11b^+^, CD115^−^, Ly6G^+^); Monocytes (CD11b^+^, CD115^+^, Ly6G^+^); Inflammatory Monocytes (CD11b^+^, CD115^+^, Ly6G^High^), Macrophages (CD11b^+^, F4/80^+^): Dendritic Cells (CD11b^+^,CD11c^+^,F4/80^−^); Alveolar Macrophages (CD11b^+^, F4/80^−^, CD115^+^, CD11c^−^); Inflammatory Macrophages (CD11b^+^/F4/80^+^, CD115^+^, CD11c^+^). **Figure S3.** Results of the multiplex cytokine Bioplex array. Multiplex bead-based ELISA was used to measure the concentration of 23 cytokines and chemokines in the BAL supernatant of β-ENaC-Tg mice and their WT littermate controls at 6 and 8 weeks old (*n* = 9). The results show significant increases in neutrophil-associated chemokines including KC, MIP-1α, MIP-1β, and G-CSF for the β-ENaC-Tg mice at either 6 weeks, 8 weeks, or both. **Figure S4.** Flow cytometry gating strategy: citrullinated histone. A representative schematic depicting the method for selecting neutrophils undergoing histone citrullination in the BAL of uninfected WT and β-ENaC-Tg mice. Single cells, negative for zombie aqua fixable viability dye were considered live. The myeloid cell marker CD11b was used to separate myeloid cells from other cell types. Neutrophils positive for citrullination were CD11b^+^, CD115^−^, Ly6G^+^, histone H3 (citrulline R2+R8+R17)^+^. **Figure S5.** Analysis of citrullination positive cell populations in the BAL of 6 and 8 week old mice. Flow cytometry of BAL cells showed significantly more neutrophils (Ly6G^+^, CD11b^+^) cells and more total citrullination (CitH3) positive cells present in the β-ENaC-Tg mice compared to WT littermate controls. There was not a significant difference between percent of citrullination positive neutrophils in the BAL (CitH3^+^, Ly6G^+^).

## Data Availability

The datasets generated and analyzed during the current study are available in the Dryad online repository: 10.5061/dryad.8pk0p2nmb

## Abbreviations

CF: Cystic fibrosis
NET: Neutrophil extracellular traps
ENaC: Epithelial sodium channel
