# Supplementary material for: Neutrophil extracellular traps are present in the airways of ENaC-overexpressing mice with cystic fibrosis-like lung disease
Source: BMC Immunol. 2021 Jan 21;22:7. doi: 10.1186/s12865-021-00397-w (PMC7819174; doi:10.1186/s12865-021-00397-w)
Supplement: Supplementary file 1 — Additional file 1: Figure S1. Genotyping of β-ENaC-Tg mice by PCR. Mice were identified as either WT C57BL/6 or heterozygous for over-expression of β-ENaC using PCR of the intron region of Scnn1b. (A) A schematic representation of the expected PCR product sizes for both WT and transgenic β-ENaC-Tg. (B) A representative gel electrophoresis comparing the final PCR product of WT and heterozygous β-ENaC-Tg mice. Figure S2. Flow cytometry gating strategy: myeloid cells. A representative schematic depicting the method for selecting myeloid cell populations present within the BAL of uninfected WT and β-ENaC-Tg mice. Single cells, negative for zombie aqua fixable viability dye were considered live. The myeloid cell marker CD11b was used to separate myeloid cells from other cell types. The CD11b+ cells were considered the parent cell population for all cell-types measured. The markers used for each cell type is as follows: Neutrophils (CD11b+, CD115−, Ly6G+), Eosinophils (CD11b+, CD115−, Ly6G+); Monocytes (CD11b+, CD115+, Ly6G+); Inflammatory Monocytes (CD11b+, CD115+, Ly6GHigh), Macrophages (CD11b+, F4/80+): Dendritic Cells (CD11b+,CD11c+,F4/80−); Alveolar Macrophages (CD11b+, F4/80−, CD115+, CD11c−); Inflammatory Macrophages (CD11b+/F4/80+, CD115+, CD11c+). Figure S3. Results of the multiplex cytokine Bioplex array. Multiplex bead-based ELISA was used to measure the concentration of 23 cytokines and chemokines in the BAL supernatant of β-ENaC-Tg mice and their WT littermate controls at 6 and 8 weeks old (n = 9). The results show significant increases in neutrophil-associated chemokines including KC, MIP-1α, MIP-1β, and G-CSF for the β-ENaC-Tg mice at either 6 weeks, 8 weeks, or both. Figure S4. Flow cytometry gating strategy: citrullinated histone. A representative schematic depicting the method for selecting neutrophils undergoing histone citrullination in the BAL of uninfected WT and β-ENaC-Tg mice. Single cells, negative for zombie aqua fixable viability dye were consid [file 12865_2021_397_MOESM1_ESM.docx]

**Neutrophil extracellular traps are present in the airways of ENaC-overexpressing mice with cystic fibrosis-like lung disease**

Samantha L Tucker^1^, Demba Sarr^1^ and Balázs Rada^1,2^

**Supplementary figures 1-5**

**
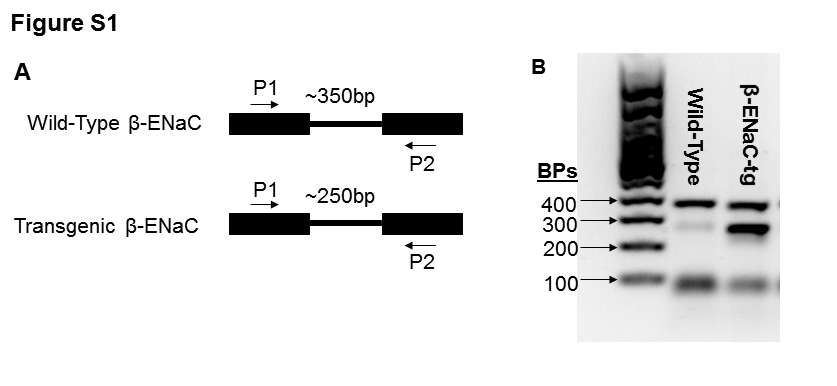
**

**
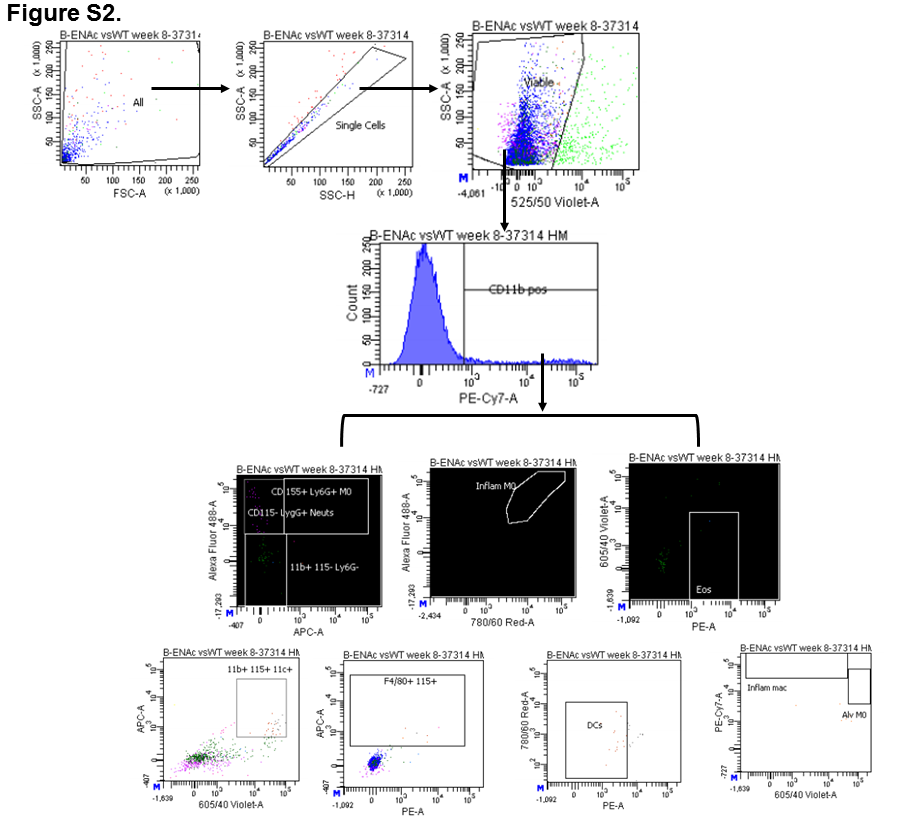
**


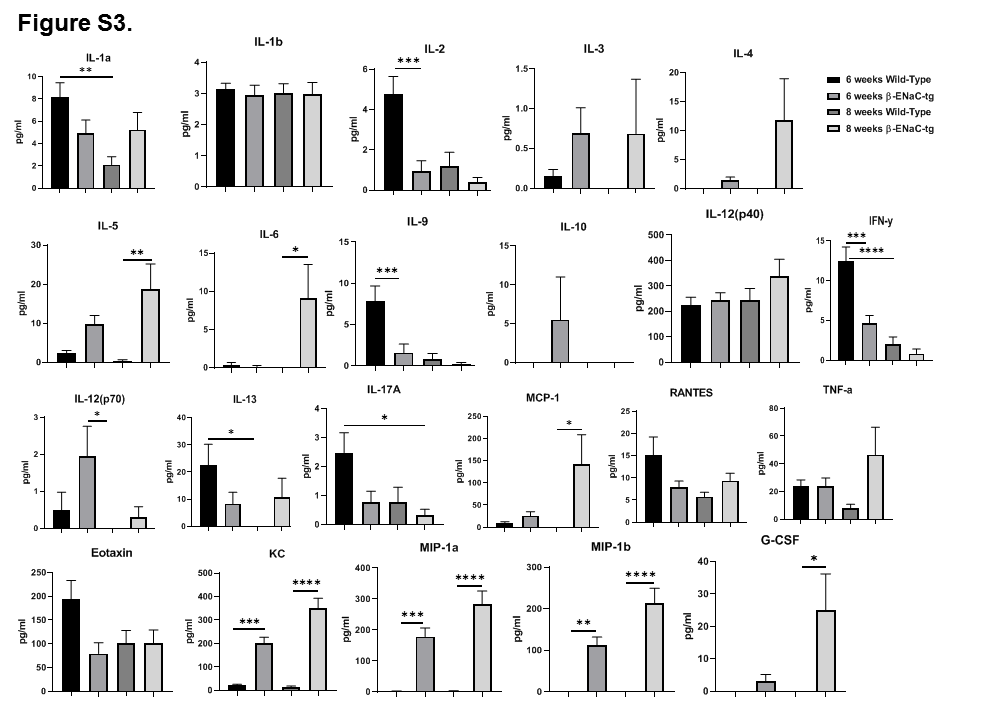


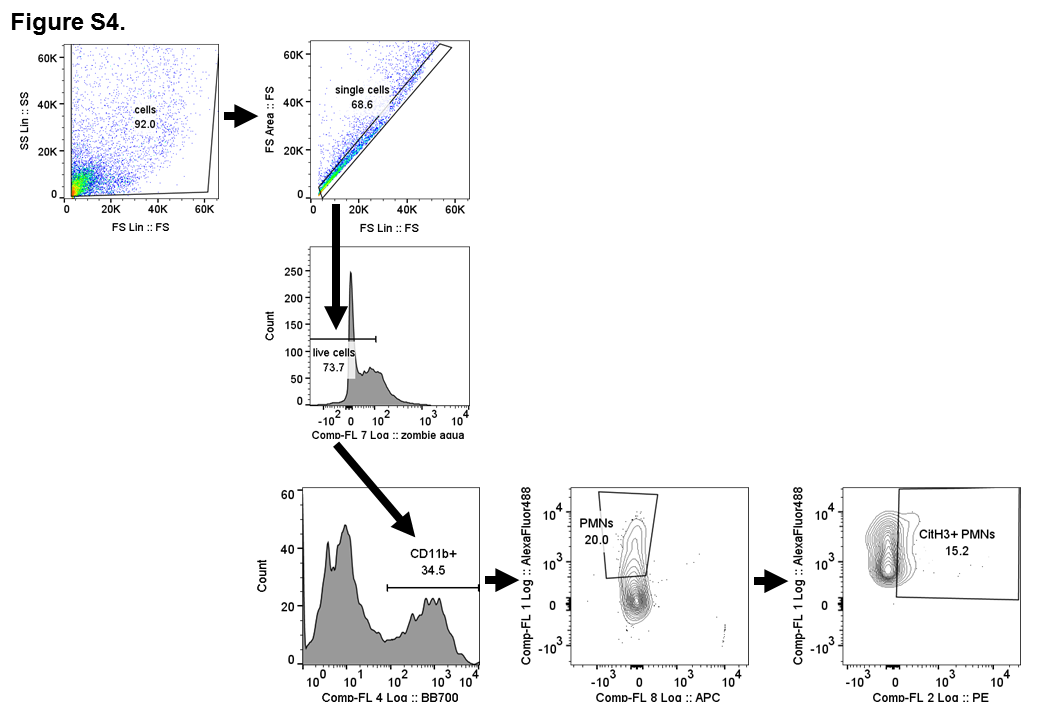


**
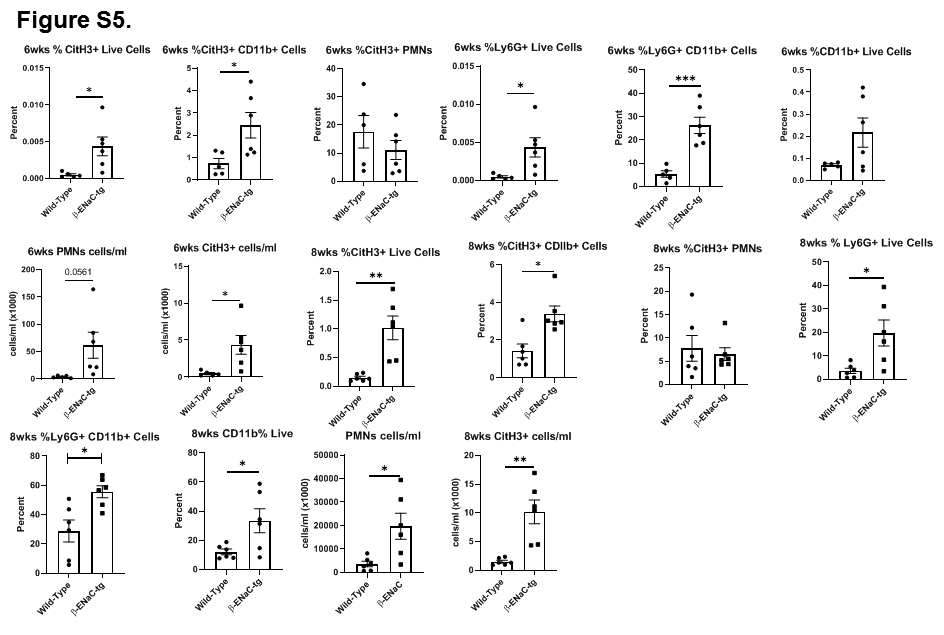
**
